# Protein arginine methyltransferases in renal development, injury, repair, and fibrosis

**DOI:** 10.3389/fphar.2023.1123415

**Published:** 2023-02-03

**Authors:** Jianjun Yu, Chao Yu, Georgia Bayliss, Shougang Zhuang

**Affiliations:** ^1^ Department of Nephrology, Shanghai East Hospital, Tongji University School of Medicine, Shanghai, China; ^2^ Department of Medicine, Rhode Island Hospital and Alpert Medical School, Brown University, Providence, RI, United States

**Keywords:** protein arginine methyltransferases, methylation, post-translational modifications, renal development, injury, repair, kidney, fibrosis

## Abstract

Protein arginine methyltransferases (PRMTs) methylate a range of histone and non-histone substrates and participate in multiple biological processes by regulating gene transcription and post-translational modifications. To date, most studies on PRMTs have focused on their roles in tumors and in the physiological and pathological conditions of other organs. Emerging evidence indicates that PRMTs are expressed in the kidney and contribute to renal development, injury, repair, and fibrosis. In this review, we summarize the role and the mechanisms of PRMTs in regulating these renal processes and provide a perspective for future clinical applications.

## Introduction

Methylation modification is an essential tool for manipulating gene expression. As the study of epigenetics has progressed in recent years, the importance of methylation modifications in regulating the pathogenesis of various diseases has been acknowledged. Methylation in epigenetic modifications primarily generates epigenetic marks such as DNA methylation, RNA methylation, and histone methylation by reversibly transferring methyl groups in chromatin (Walker and Burggren, 2020). Histone methylation is predominantly found in the arginine and lysine amino acid residues on histone tails and is essential for maintaining histone structure and modulating transcriptional processes.

Arginine methylation is regulated by protein arginine methyltransferases (PRMTs) and protein arginine demethylases. PRMT is a large family of proteins with distinct N-terminal sequences. Depending on the number of transferred methyl groups and the guanidine position of the arginine terminal, PRMTs are categorized into four types, type I (PRMT 1,3,4,6 and 8), type II (PRMT 5 and 9), Type III (PRMT7) and Type IV (PRMT2). PRMTs can cause monomethylation and dimethylation of arginine residues, resulting in the generation of three forms of methylarginine: ω-NG-monomethylarginine (MMA), ω-NG, NG-asymmetric dimethylarginine (ADMA), and ω-NG, N′G-symmetric dimethylarginine (SDMA). Type I PRMTs catalyze the production of MMA and ADMA; type II PRMTs catalyze production of MMA and SDMA, and type III (PRMT7) exclusively catalyzes MMA production. Type IV (PRMT2) has only been described in studies with yeast (internal guanidinium methylation) (Nott et al., 2015). Thus, the production of ADMA and SDMA usually indicates the existence of PRMTs. MMA, ADMA, and SDMA in histones are commonly related to transcriptional activation and gene silencing (Mosammaparast and Shi, 2010), and increased circulated ADMA can inhibit NOS (NO synthase), which then inhibits NO (Nitric oxide) production and diminishes endothelial protection (Couto et al., 2020).

Many studies have shown that the PRMT family is also involved in the arginine methylation of non-histone proteins. Many transcription factors, coactivators, and other proteins are subject to methylation (Lee and Stallcup, 2009). In addition to controlling gene expression, PRMTs-mediated methylation participates in a variety of biological processes such as proliferation, differentiation (Adli et al., 2015), transcription (Liu et al., 2020a), variable splicing (Braun et al., 2017), signal transduction (Xu and Richard, 2021), cell cycle (Schneider et al., 2021), and apoptosis (Diao et al., 2019a). PRMTs also affect phenotypic changes and are involved in numerous physiological and pathological processes, such as embryonic development (Vougiouklakis et al., 2017), inflammatory response (Kim et al., 2016), and tumorigenesis (Hwang et al., 2021). As a consequence, the PRMT family has been linked to the development of various diseases and dysfunctions in a multitude of organs, including the heart (Couto et al., 2020), liver (Zhao et al., 2019), lung (Lim et al., 2013), and kidney (Zhang and Zhuang, 2020).

Increasing evidence revealed that PRMTs are closely associated with renal development, acute and chronic renal injury and repair, fibrosis and other renal pathology like renal cell carcinoma (Liu et al., 2015; Liu et al., 2020b), diabetic nephropathy (Park et al., 2017), and renal transplant rejection (Zakrzewicz et al., 2011). In this article, we summarize the role and mechanism of the PRMTs in regulating renal development, injury, repair, and fibrosis and provide some insight for future research and development of PRMT inhibitors and their clinical application.

## PRMT activation and their substrates

PRMTs exert their biochemical effects *via* methylating various histone and non-histone substrates. Additionally, the methylation process of PRMTs seems to be substrate-dependent: different members can methylate the same substrates, and multiple PRMTs can mediate the same substrates (Table 1).

**TABLE 1:** The histone methylation site of PRMTs.

PRMTs types	Member	Histone site	Function
Ⅰ	PRMT1	H4R3	Transcription activation Nott et al. (2015); Walker and Burggren (2020)
		H2AR3, R11, R29	Transcription repression Mosammaparast and Shi (2010)
	PRMT2	H3R8	Transcriptional activation Couto et al. (2020)
		H4	Transcription activation Lee and Stallcup (2009)
	PRMT3	H4R3	Transcriptional activation Adli et al. (2015)
	PRMT4/CARM1	H3R2	Transcriptional activation Liu et al. (2020a)
		H3R17, R26	Paternal genome reprogramming Braun et al. (2017)
			Embryo development Xu and Richard (2021)
		H3R42	Transcriptional activation Schneider et al. (2021)
	PRMT6	H2AR3, R29	Transcription repression Mosammaparast and Shi (2010)
		H3R2	Transcriptional activation Diao et al. (2019a)
		H3R42	Transcriptional activation Schneider et al. (2021)
		H4	Unknown
	PRMT8	H4	Unknown
Ⅱ	PRMT5	H2AR3	transcriptional repression Vougiouklakis et al. (2017)
		H3R2	maintain euchromatin Kim et al. (2016)
		H3R8	Transcription repression Hwang et al. (2021)
		H4R3	Transcription repression Hwang et al. (2021)
Ⅲ	PRMT7	H2AR3	Derepress DNA repair Zhao et al. (2019)
		H3R2	maintain euchromatin Kim et al. (2016)
		H4R3	Transcription repression Lim et al. (2013)
		H2BR29, R31, R33	stabilizes nucleosome structure Zhang and Zhuang (2020)
		H4R17	Promote spermatogenesis Lim et al. (2013)
		H4R19	Unknown

Type I PRMTs are broadly expressed in mammalian tissues and organs, with particular spatial and temporal expression (Wu et al., 2021). Further subcellular localization showed that the methylation substrates of type I PRMTs aggregate in the cell membrane, cytoplasm, and nucleus, indicating that Type I PRMTs can function in diverse organelles. Physiologically, Type I PRMTs play a regulatory role in the development, aging, and regulation of the body’s internal environment by methylating histones and non-histone substrates. The histone substrates are primarily concentrated in histones H3 and H4 (Tewary et al., 2019). For example, PRMT1 (Chen et al., 2019), PRMT3 (Lei et al., 2022), and PRMT8 (Dong et al., 2021) can induce methylation of H4R3 and modulates transcriptional activity. PRMT6 is the primary enzyme responsible for H3R2. By competing for H3K4me3, H3R2me2a can inhibit the gene transcriptional activity (Guccione et al., 2007). Furthermore, PRMT2 and PRMT4 catalyze the methylation of arginine 8 (H3R8me2a) on histone H3 and affect normal and abnormal cell cycle progression (Dong et al., 2018; Hu et al., 2020). PRMT4 appears to prioritize methylation of histone H3R2, R17, and R26 (30), and this methylation is involved in regulating numerous biological responses, including embryonic development (Hatanaka et al., 2017; Yang et al., 2019), transcriptional activation (Yu et al., 2020), chromatin remodeling (Dacwag et al., 2009), cell regeneration (Sarker et al., 2019), and cellular autophagy (Yu et al., 2020).

PRMT5 is a well-studied type II PRMT. It is abundantly expressed in the cytoplasm and nucleus of the lungs, liver, heart, kidneys, and other tissues (Wu et al., 2021). Except for H2B, all histones (but mainly H2R3, H3R2, H3R8, and H4R3) are the substrates of PRMT5. The primary function of PRMT5 is to repress gene transcription (Fabbrizio et al., 2002) and to regulate cell proliferation, pluripotency, and differentiation. For example, PRMT5 can inhibit mesenchymal stem cells (MSCs) development into osteoblasts by reducing the enrichment of H3R8me2s and H4R3me2s in the promoter region (Kota et al., 2018).

PRMT7 is the representative enzyme of type III PRMTs and the sole isoform of PRMTs that can catalyze arginine monomethylation. PRMT7 is mainly expressed in the lung (Cheng et al., 2018), brain tissue (Lee et al., 2019), breast (Liu et al., 2020c), and other tissues with subcellular localization in the cytoplasm. PRMT7 can bind all known nucleosome histones (Zurita-Lopez et al., 2012). H2BR29, R31, R33, H4R17, and R19 were all methylation sites of PRMT7 (Feng et al., 2013). PRMT7 is also engaged in a variety of biological processes, including DNA damage repair, variable shearing, cell proliferation, and male reproduction (Wang et al., 2021). For example, the H4R19me has an imprinting function in the development of male embryos (Jelinic et al., 2006). In addition, PRMT7 may bind to arginine residues in an alkaline environment and is associated with RNA splicing variability and cancer cell proliferation (Li et al., 2021).

## PRMTs in renal development

The kidney goes through a complicated development process originating in the intermediate mesoderm (IM). It proceeds through three stages: the pronephros (E8 in mice), the mesonephros (E9 in mice), and the metanephros (E10.5 in mice) (Dressler, 2009; Reidy and Rosenblum, 2009). The pronephros and mesonephros are transient organs that degrade throughout embryonic development, whereas the metanephros matures into a permanent kidney (Schedl and Hastie, 2000). At the end of the mesonephros, the ureteral bud (UB) extends from the terminal portion of the mesonephric tubules and continues into the metanephric mesenchyme. And the UB and the metanephric mesenchyme (MM) mutually stimulate the production of cap mesenchyme (CP) and renal vesicles (RV) (Little et al., 2007). Subsequently, these structures further produce functional cells, such as nephrons (including glomerular, proximal tubular, and distal tubular epithelial), vasculature (Lopez and Gomez, 2011), interstitial cells, mesangial cells, and pericytes in the kidney (Kobayashi et al., 2014), as well as a complete urinary tubule system.

Growing evidence suggests that PRMT activation is closely linked to kidney development. According to an analysis of total proteome mass spectrometry study, PRMT1, 4, and 5 are expressed in embryonic kidney tissue (Kim et al., 2014; Stopa et al., 2015). While PRMT4 is active only in early fetal development, the expression of PRMT1 and 5 is still detectable at 6 months (Hong et al., 2012). Thus, PRMT1 and PRMT5 may be essential in kidney development and maintenance. Different expression patterns of PRMTs in developmental stages of the kidney imply that they may play different roles in various developmental stages. The roles of PRMT members in kidney development are summarized in Table 2.

**TABLE 2:** The role of PRMTs in renal development.

PRMT numbers	Histone modification	Functions and mechanisms	References
CARM1	H3R2me2a	Involved in differentiation of early nephrons and CP, immature kidney and collecting duct by methylation of NICD	Liu et al. (2015), Liu et al. (2020b)
	H3R17me2a	Participate in the differentiation of the cap mesenchyme in its early nephrogenesis collaborating with SIX2	Liu et al. (2015), Liu et al. (2020b)
PRMT5	H3R8me2s	Engage in UB development through E-cadherin together with SIX2	Liu et al. (2015)
PRMT6	H3R2me2a	Co-expressed with Pax2 methylation at early stages of kidney development; Engaged in the regulation of the entire metanephros	Liu et al. (2015), Park et al. (2017)

CP, cap mesenchyme; NICD, notch intracellular domain; PRMT, protein arginine methyltransferase; UB, ureteric bud.

H3 histone markers for arginine methylation are highly expressed during the development of the metanephros. H3R2me2 and H3R17me2 markers were significantly expressed in cap mesenchyme, H3R2me2 and H3R8me2 were highly expressed in immature kidney and collecting duct, and H3R8me2 was substantially expressed in immature nephron (McLaughlin et al., 2014). However, it is currently uncertain which PRMT is responsible for inducing the methylation of H3 histones. It has been reported that H3R2me2 and H3R17me2 are co-located with Pax2 and Six2 in the CP, whereas H3R8me2 is co-located with Pax2 and E-cadherin in the UB structure, according to *in situ* section immunofluorescence analysis. In general, PRMT4 catalyzes the formation of H3R2me2a and H3R17me2a, PRMT5 catalyzes the formation of H3R2me2s and H3R8me2s, and PRMT6 can only create H3R2me2a. As such, it is speculated that PRMT4 may play a role in the development of CP by methylating H3R2 and H3R17, PRMT5 may be required for the development of UB by producing H3R8me2s, and PRMT6 plays a crucial part in the development of the whole early kidney by methylating H3R2. In most organisms, the Notch pathway plays a significant role in early embryonic development and stem cell differentiation (Andersson et al., 2011). Previous research has demonstrated that Notch pathway proteins are engaged in promoting epithelial nephrons (Lin et al., 2014), mesangial cells (Boyle et al., 2014), and renal vascular systems (Mohamed and Sequeira-Lopez, 2019) during renal development. Coincidentally, the production of the Notch intracellular domain (NICD) happens to be the non-histone substrate of PRMT4 (Hein et al., 2015). Thus, PRMT4 may be engaged in early nephron cap mesenchymal differentiation by producing methylation markers in the Arg-2, Arg-17 of histone H3, and the C-terminal of NICD, which is correlated to podocytes, mesangial cells, and angiogenesis.

Furthermore, a crosstalk between arginine methylation and lysine methylation modifications allows for fine-tuning of the kidney development process. Immunofluorescence staining demonstrated that histone H3K4me3 is abundant in CP and nascent nephrons (McLaughlin et al., 2014). In addition, the presence of H3R2me2a in the mammalian promoters is consistent with that of H3K4me3 (Guccione et al., 2007), and H3R2 methylation and H3K4 methylation are mutually antagonistic, as PRMT6-mediated H3R2me2a blocks H3K4me3 mediated by the ASH2/WDR5/MLL family methyltransferase complex at the tail end of histone H3. Further investigations have revealed that H3Rme2 and Pax2 are co-expressed throughout the early stages of kidney development (McLaughlin et al., 2014) and that the increase in H3K4me3 is closely associated with Pax2 gene deletion. Consequently, PRMT6 may be involved in regulating the whole metanephros (including the renal tube, nephron, renal interstitium, and so on), albeit the specific mechanism remains to be further investigated.

## PRMTs in renal injury

### The role of PRMTs in acute kidney injury

Acute kidney injury is defined by a sudden fall in the glomerular filtration rate and a decline in urine volume. Ischemia, toxins, sepsis, and renal obstruction are major causes of acute kidney damage (Tang and Zhuang, 2015). The primary pathophysiological changes in AKI are apoptosis and necrosis of renal tubular epithelial and endothelial cells. Recent studies have shown that PRMTs are also involved in the pathogenesis of AKI from varied causes. Renal PRMT5 expression rose 12 h after renal ischemia and remained elevated at 24 h (Diao et al., 2019b), whereas renal PRMT1 expression levels were not significantly altered following ischemia reperfusion (IR) (Nakayama et al., 2014). Immunostaining showed that PRMT5 was mainly expressed in epithelial cells of proximal tubules in the renal cortex. This was further verified in cultured HK cells, a human proximal tubular cell line, in which hypoxia/reoxygenation induced expression of PRMT5. PRMT5 upregulation was accompanied by inflammasome formation. Inflammasomes mediate the generation and release of pro-inflammatory substances such as mature interleukin-1β (IL-1β) and eventually cause pyroptosis (Tavakoli Dargani and Singla, 2019). Inhibiting PRMT5 with EPZ (a selective inhibitor) or siRNA can increase the Nrf2/HO-1 signal pathway, minimize ROS-induced apoptosis and inflammation, decrease renal injury, and accelerate tubular cell proliferation. These results suggest that PRMT5 activation contributes to the pathogenesis of AKI and inadequate generation of renal tubular epithelial cells during recovery. Nevertheless, the role of PRMT5 in AKI needs to be verified by using PRMT5 knockout mice.

ADMA expression appears to have a strong influence on oxidative stress in AKI. ADMA and SDMA levels increased in the ischemia/reperfusion model of the rat kidney due to a pathological shortage of L-arginine (Betz et al., 2013). ADMA accumulation and high expression of PRMT1 in the kidney are consistent with renal capillary loss and tubular cell necrosis (Nakayama et al., 2014). ADMA suppresses the generation of eNOS and NO and promotes renal function deterioration. It is speculated that ADMA production may be mediated by other PRMT members rather than PRMT1 since PRMT1 is not significantly elevated in the early stage of AKI. Although PRMT1 was not significantly increased in the IR model, PRMT1 expression was considerably upregulated in acute liver injury (Zhao et al., 2019) and acute lung injury (Lim et al., 2013), strongly related to oxidative stress-induced apoptosis. Thus, PRMT1 expression may be time and tissue/organ-dependent. Currently, studies on PRMTs in AKI are only limited to IR injury models; other models (e.g., nephrotoxic drug models, sepsis models, rhabdomyolysis models, etc.) should be used to demonstrate their roles in AKI.

### The role of PRMTs in chronic kidney disease

Multiple systemic disorders like diabetes and hypertension might result in permanent kidney impairment—chronic kidney disease. In contrast to AKI, persistent kidney injury causes more implicated pathophysiological responses as it becomes chronic kidney disease. It triggers capillary loss due to pericyte shedding, immune-inflammatory cell infiltration, interstitial fibrosis, mesangial cell apoptosis, etc., (Sato et al., 2020; Tanemoto and Mimura, 2022). Some members of the PRMT family are also involved in this process. It has been reported that high PRMT7 expression was associated with a lower risk of persistent kidney injury caused by antibiotics (Zheng et al., 2005), and PRMT1 expression was upregulated in renal epithelial cells of DN mice (Chen et al., 2019) as well as in podocytes of patients with DN (Zhu, 2018). Furthermore, the elevation of ADMA was positively associated with the degree of chronic kidney injury in a rat chronic kidney model (Ueda et al., 2009).

Long-term hypertension is also linked to persistent renal injury and chronic kidney disease (Whelton et al., 2017), Upregulation of PRMT1 and PRMT3 in the kidney, as well as downregulation of DDAH expression, were shown to be associated with an increase in ADMA in the rat 5/6 nephrectomy model, exacerbating chronic kidney injury by damage to vascular endothelium (Matsuguma et al., 2006). According to Okubo et al., increased expression of ADMA might enhance vascular endothelial dysfunction by downregulating NO in a canine model of sustained kidney injury. This shift could be mitigated by treatment with adenosine dialdehyde (a PRMT inhibitor) (Okubo et al., 2005). Although type I PRMTs are known to produce ADMA, the precise PRMT members and the mechanism of ADMA-induced damage of the vascular endothelium have yet to be reported.

High lipid-induced PRMT1 expression may also be associated with persistent renal injury. Hyperglycemia and hyperlipidemia are important causative factors of diabetic kidney injury (Hung et al., 2021). Excessive hyperglycemia can activate protein kinase RNA-like endoplasmic reticulum kinase (PERK) and transcription factor 6 (ATF6) by upregulating H4R3me2a, promoting EMT and ER stress, accelerating PTEC apoptosis, and aggravating renal fibrosis by upregulating PRMT1 expression (Chen et al., 2019). In another report, high, lipid-induced PRMT1 expression was also demonstrated to mediate apoptosis in mesangial cells *via* the PERK and ATF6 pathways (Park et al., 2017), and inhibition of PRMT1 expression can delay the progression of diabetic nephropathy by suppressing the AGE-RAGE-mediated rise in ADMA (Leiper, 2005). Furthermore, PRMT1 expression was elevated in the renal cortex in a 5/6 nephrectomy rat model, which was consistent with overexpression of nNOS and ADMA accumulation; NO synthesis may be affected by nNOS-α and nNOS-β, which may exacerbate renal damage and promote progression of CKD (Tain et al., 2011). As a result, further mechanistic studies are needed to clarify the role and mechanism of PRMTs in persistent renal injury.

## PRMTs in repair

The kidney has a remarkable but limited ability to repair and regenerate. Following kidney injury, the self-protection mechanism is quickly initiated. When the kidney is injured slightly, surviving proximal tubular cells can undergo dedifferentiation, redifferentiation, self-proliferation, and repair (Guo et al., 2019). Endothelial cells regenerate, and kidney function maintains. However, when kidney was injured severely or persistent (e.g., chronic ischemia or hypoxia), tubular cells would experience cellular necrosis and apoptosis, partial epithelial mesenchymal-transition (EMT), and G2/M cell cycle arrest, capillaries are shed, renal interstitial fibrosis develops (Kumar, 2018).

PRMT5 is closely connected to kidney recovery following an acute injury Braun et al. (2004). Found that PRMT5 expression in the renal tubular epithelium was increased within 24 h and remained high after peaking in an *in vitro* IR model in mice. According to immunohistochemistry, PRMT5 was significantly expressed in the proximal tubular epithelium in the kidney’s inner cortical and outer medullary sections. *In vitro* experiments of tubule formation, PRMT5 was also increased in human proximal tubule cells that differentiated into tubule-like structures in the Matrigel matrix (Braun et al., 2004), suggesting the importance of PRMT5 in facilitating the renal repair and tubular regeneration after renal injury. Apart from tubular epithelial cells, PRMT5 expression is also enhanced in endothelial cells in zebrafish models (Quillien et al., 2021), and implicated in angiogenesis by regulating the transcription of ETS transcription factors and adhesion proteins in endothelial cells. Thus, it’s worth investigating whether PRMT5 plays a role in vascular repair after kidney injury. Currently, the possible mechanisms of PRMT5 in renal tubular regeneration have yet to be studied and need to be studied.

After a persistent renal injury, PRMT1 was increased in both kidneys of rats, which was associated with higher ADMA in plasma and kidneys in a 2K1C (two-kidney, one-clip) model. In contrast, nebivolol decreased the expression of PRMT1, ADMA, and DDAH2 and lessened renal fibrosis and capillary damage, and improved renal function; the exact mechanism is not known (Wang et al., 2018). In another study, the pharmacological reduction of ADMA improved endothelial function and reduced ischemic injury in the kidney (Lee et al., 2021). Given that NO is a vasoprotective factor and able to induce dilation of vascular smooth muscle, the ADMA-NO axis may help prevent kidney failure by enhancing endothelial repair.

## PRMTs in fibrosis

A growing body of evidence shows that PRMTs play an important role in the myofibroblast activation and progression of renal fibrosis *via* mechanisms involving microvascular oxidative stress, EMT, ECM (extracellular matrix) deposition, and inflammatory response (Liu, 2011).

Our recent studies showed that expression levels of PRMT1 and its methylation marker, H4R3Me2a, were increased in the kidney following UUO injury (Zhu et al., 2020). Administration of AMI-1, a selective inhibitor of PRMT1, inhibited expression of PRMT1 and H4R3Me2a, attenuated extracellular matrix protein deposition, and inhibited renal fibroblast activation and proliferation in the injured kidneys. In line with these observations, treatment with AMI-1 or siRNA, specifically silencing of PRMT1, also suppressed TGF β1-induced expression of α-SMA and collagen I in cultured renal interstitial fibroblasts, which corresponded to decreased expression of PRMT1 and H4R3me2a. Furthermore, PRMT1 inhibition suppressed activation of the TGF-β/Smad3 signaling *in vivo* and *in vitro*. These data suggest that PRMT1 activation contributes to renal fibrogenesis in the model of obstructive nephropathy. In support of this study (Chen et al., 2019), demonstrated that PRMT1 induces ER stress and epithelial-mesenchymal transition in renal tubular epithelial cells and contributes to diabetic nephropathy. One of the critical findings in this study is that overexpression of PRMT1 in HK2 cells exposed to high glucose medium resulted in decreased expression of E-cadherin and β-catenin (epithelial markers) and increased expression of ZEB-1, fibronectin, and α-SMA (mesenchymal markers), whereas the downregulation of PRMT1 had the opposite results (Chen et al., 2019). In addition, the profibrotic role of PRMT1 was also observed in lung and liver tissue (Zakrzewicz et al., 2015). Observed that expression of PRMT1 was increased in IPF (idiopathic pulmonary fibrosis) lung fibroblasts and IPF lungs and in lungs of bleomycin-treated mice, and PRMT1 knockdown or inhibition of PRMT activity reduced IPF fibroblast motility (Yan et al., 2022). Found that the expression of PRMT1 was elevated in cirrhotic livers from human patients and in fibrotic livers of the mouse models, and the application of a selective inhibitor of PRMT1, PT1001B, or hepatic stellate cells (HSC)-specific PRMT1 knockout attenuated HSC activation and liver fibrosis in fibrotic models.

In contrast to the profibrotic role of PRMTs observed in above studies (Wu et al., 2019), demonstrated that inhibition of PRMT1 with PT1001B exacerbated the fibrotic injury in the kidney of mice following UUO and promoted expression of profibrotic proteins in cultured renal fibroblasts. The discrepancy between the results obtained from these studies remains unclear. Recently, the work from the same group also suggests that PRMT3 exerts an anti-fibrotic effect in the kidney, as illustrated by elevated PRMT3 in the kidney of mice subjected to either UUO or FA (Wang et al., 2022); either homozygous or heterozygous deletion of the *Prmt3* gene increased expression levels of α-SMA and collagen I in injured kidneys.

In summary, the role of PRMTs in renal fibrosis is still controversial. Further research using knockout mice and more specific inhibitors is needed to clarify their roles in this pathological process.

## Conclusion and perspectives

**FIGURE 1:**
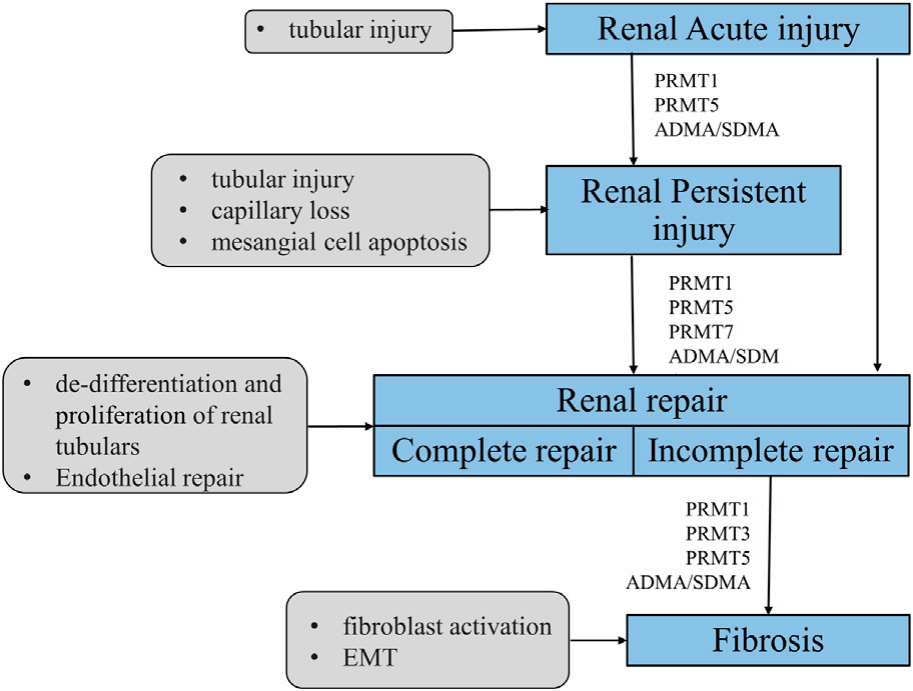
Mechanism of PRMTs-mediated renal injury, repair and renal fibrosis. After a minor injury, the kidney can repair itself (complete repair); however, when severe injury or injury persists, the self-repair mechanism of the kidney cannot resist the external damage, it will tend to develop fibrosis (incomplete repair). PRMTs are widely involved in the process of kidney injury, repair and fibrosis. PRMT1 and ADMA/SDMA are involved in acute and chronic kidney injury, repair and fibrosis. PRMT5 mainly plays a role in acute kidney injury and repair, and PRMT7 is currently described only in the repair process.

There is mounting evidence that PRMTs play an important role in kidney development, damage, repair, and fibrosis (Figure 1). They are also linked to oxidative stress, cell necrosis, apoptosis, cell cycle arrest, EMT, and immune-inflammatory response. However, the research on PRMTs in kidney diseases is still relatively limited. Currently, most studies are only focused on PRMT1 and PRMT5, with few studies on other members (Table 3). The types and quantities of PRMT inhibitors are limited and lack selectivity; genetic techniques for overexpression or silencing (e. g., knockout mice and transfected cells) are lacking. As a result, not only further experimental research on the specific mechanisms of different PRMTs in various renal disease models needs to be carried out, but also further work is required to develop more effective and specific PRMT inhibitors.

**TABLE 3:** The role of PRMTs in renal injury, repair and fibrosis.

Pathological processes	Targets	Models	Functions and mechanisms	References
AKI	PRMT5	HK-2cells; I/R induced AKI	Downregulate Nrf2/HO-1; Increases ROS-induced apoptosis and inflammation; Inhibit tubule cell proliferation	Zakrzewicz et al. (2011)
	PRMT1,ADMA	I/R induced AKI	Suppress the production of eNOS and NO, Resulte in necrosis of renal capillaries and tubular cells	Tewary et al. (2019), Wu et al. (2021)
CKI(DN)	PRMT1	rat mesangial cell line	Mediate lipotoxicity-induced apoptosis in mesangial cell; Activate ER stress	Chen et al. (2019)
	PRMT1	PTEC cell line, HK2 cells	Induce ER stress and apoptosis through activating PERK and ATF6; Activate EMT activation; Alleviate both tissue injury and renal fibrosis	Wu et al. (2021)
CKI(Hypertension)	ADMA	5/6Nx	Reduce NO production	Lei et al. (2022)
			Enhance vascular endothelial dysfunction	
Repair	PRMT5	RPTEC; IRI	Enhance the proliferation of tubular cells	Dong et al. (2021)
	PRMT1	2K1C rat model	Prohibit NCK production of ROS and ADMA by suppressing PRMT1 expression to protect the kidney	Guccione et al. (2007)
	ADMA	I/R Model with SD rats	Promote endothelial function recovery and decrease renal inflammatory response by suppressing DDAH	Hu et al. (2020)
Fibrosis	PRMT1	NRK-49F and HK2 cells; UUO	Reduce H4R3me2a expression, Decrease ADMA production, increase NO, and promote renal fibrosis through blockade of PRMT1	Dong et al. (2018)
	PRMT1	UUO	Downregulate DDAH, increased ADMA and reduced renal fibrosis *via* blocking PRMT1	Qi et al. (2002)
	PRMT1	UUO; NRK-49F cells	Reduce renal fibroblast activation by downregulating TGF-β/Smad3 pathway to attenuate renal fibrosis with inhibitating PRMT1	Yang et al. (2019)
	PRMT3	UUO, FA	Increase H4R3me2a expression	Hatanaka et al. (2017)

2K1C, two-kidney, one-clip rat model; ADMA, asymmetric dimethylarginine; AKI, acute kidney injury; ATF6, activating transcription factor 6; BTG1, B-cell translocation gene 1; ccRCC, clear cell renal cell carcinoma; DDAH, dimethylarginine dimethylaminohydrolase; DN, diabetic nephropathy; EMT, epithelial-to-mesenchymal transition; eNOS, endothelial nitric oxide synthase; ER, endoplasmic reticulum; H4R3me2a, histone H4 arginine 3 asymmetric demethylation; HK2, human kidney 2; IRI, renal ischemia with reperfusion injuries; I/R, ischemia/reperfusion; NCK, non-clipped kidney; Nrf2/HO-1, NF-E2-related factor/heme oxygenase-1; NO, nitric oxide; NRK-49F, normal rat kidney 49F cells; Nx, 5/6 nephrectomy; PERK, protein kinase RNA-like endoplasmic reticulum kinase; PRMTs, Protein arginine methyltransferases; ROS, reactive oxygen species; RPTEC, human renal proximal tubule epithelial cells; SD, Sprague-Dawley; SREBP1, sterol regulatory element-binding protein 1; TGF-β1, transforming growth factor; UUO, unilateral ureter ligation.

So far, there have been few specific investigations on the substrates of arginine methylation in the kidney. Therefore, further digging into the histone methylation sites and substrates correlated to kidney illness is also required. Given the prevalence of crosstalk between HKMTs and PRMTs, as well as between PRMTs’ family members, understanding those interactions and exact methylation sites will help us better understand disease pathogenesis and provide a theoretical framework for the development of specific targeted inhibitors.
